# Tree Biomass Estimation of Chinese fir (*Cunninghamia lanceolata*) Based on Bayesian Method

**DOI:** 10.1371/journal.pone.0079868

**Published:** 2013-11-20

**Authors:** Xiongqing Zhang, Aiguo Duan, Jianguo Zhang

**Affiliations:** State Key Laboratory of Tree Genetics and Breeding, Key Laboratory of Tree Breeding and Cultivation of the State Forestry Administration, Research Institute of Forestry, Chinese Academy of Forestry, Beijing, People's Republic of China; Manchester University, United Kingdom

## Abstract

Chinese fir (*Cunninghamia lanceolata* (Lamb.) Hook.) is the most important conifer species for timber production with huge distribution area in southern China. Accurate estimation of biomass is required for accounting and monitoring Chinese forest carbon stocking. In the study, allometric equation 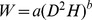 was used to analyze tree biomass of Chinese fir. The common methods for estimating allometric model have taken the classical approach based on the frequency interpretation of probability. However, many different biotic and abiotic factors introduce variability in Chinese fir biomass model, suggesting that parameters of biomass model are better represented by probability distributions rather than fixed values as classical method. To deal with the problem, Bayesian method was used for estimating Chinese fir biomass model. In the Bayesian framework, two priors were introduced: non-informative priors and informative priors. For informative priors, 32 biomass equations of Chinese fir were collected from published literature in the paper. The parameter distributions from published literature were regarded as prior distributions in Bayesian model for estimating Chinese fir biomass. Therefore, the Bayesian method with informative priors was better than non-informative priors and classical method, which provides a reasonable method for estimating Chinese fir biomass.

## Introduction

Chinese fir (*Cunninghamia lanceolata* (Lamb.) Hook.), a fast growing evergreen coniferous tree, is one of the most important tree species for timber production in southern China. As an important native tree, Chinese fir has been widely planted extending over more than 1000 years [1]. It produces excellent quality timber, with straight shape, high resistance of bending and cracking, and easily processing trait. Because of its high commercial value, the planting area of Chinese fir in China is around 9.215 million ha, accounted for 28.54% of all forested land [2]. Currently, it is thought that this conifer tree will be able to bring great profit of biomass production.

The estimation of tree biomass is needed for both sustainable planning of forest resources and for studies on the energy and nutrients flows in ecosystems. Hall [3] reviewed that the potential role of biomass is an energy source in the 21^st^ century. In addition, the United Nations Framework Convention on Climate Change and in particular the Kyoto Protocol recognize the importance of forest carbon sink and the need to monitor, preserve and enhance terrestrial carbon stocks, since changes in the forest carbon stock influence the atmospheric CO2 concentration [4]. The reliability of the forest carbon stock estimates and the understanding of ecosystem carbon dynamics can be improved by biomass equations [5], [6]. Since collecting biomass data is costly and time-consuming, accurate estimation of biomass is required for accounting and monitoring carbon stock. The biomass equations can be applied directly to tree level inventory data (diameter, height).

For estimating tree biomass for Chinese fir, many models were developed, especially for allometric equations: 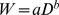, and 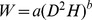 (W: tree biomass, *D*: diameter at breast height, *H*: tree height). The variable *D* ^2^ *H* was usually used in biomass equations and gave good estimates. Lin et al. [7] developed biomass model of Chinese fir with the two equations, and found that the second equation with 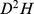 was better than the first one. Tian et al. [8] estimated the stem biomass, branch biomass, root biomass and foliage biomass of a second generation Chinese fir plantation with 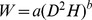, and the correlation coefficients of the models were more than 0.97. However, as tree allometry is influenced by both environmental and competitive factors [9], [10], temporal changes in these conditions are likely to affect the biomass estimation. A major limitation of these equations is that produces very different results when applied to different stands where the equations were originally developed [11].

Bayesian inference is an alternative method of statistical inference that is frequently being used to evaluate ecological models [12], [13]. Although Bayesian and classical approaches have been debated on the philosophical level in many scientific fields [14], [15], it has been shown that the Bayesian method has unique advantages in main two situations. Firstly, Bayesian methods are fully consistent with mathematical logic, while classical methods are only logical when making probabilistic statements about long-run averages obtained from hypothetical replicates of sample data, not hypotheses [16], [17]. Secondly, relevant prior knowledge about the data can be incorporated naturally into Bayesian analyses whereas classical methods ignore the relevant prior knowledge other than the sample data [15].In forestry, Bayesian methods have been adopted in several applications such as diameter distribution [18], [19], tree growth [20], individual tree mortality [21], [22], tree foliar dry matter [23], stand-level height and volume growth models [24], [25], and stand basal distribution [26]. Zapata-Cuartas et al. [27] used Bayesian method to estimate aboveground tree biomass, and obtained a reliable result.

The objective of the study was to estimate stem biomass, branch biomass, foliage biomass, and root biomass of Chinese fir using 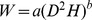 based on a Bayesian framework. In addition, the Bayesian method was compared with the classical method for biomass estimation of Chinese fir.

## Materials and Methods

### Study Site

**Figure 1:**
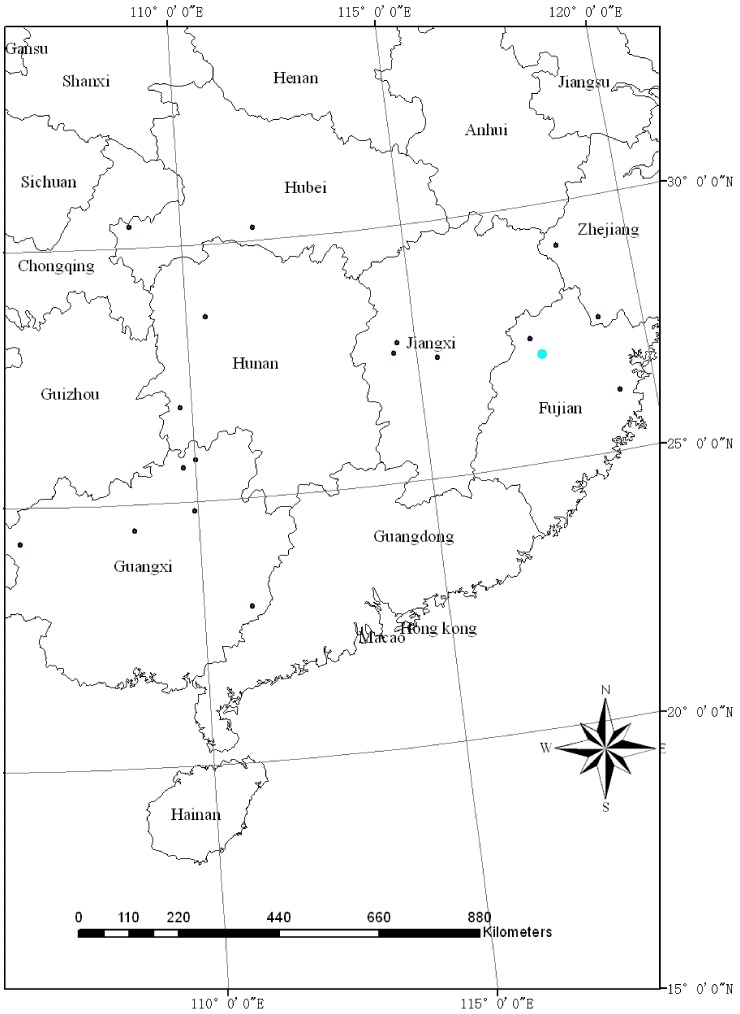
Geographical location of Chinese fir compiled in the published literature. The big blue dot is the location for the study site. Other black dots are the locations of published literature for studying Chinese fir biomass.

The plantations studied were at Weimin farm, Shaowu city (27.08°N, 117.72°E), in Fujian Province, southern China (Fig. 1) which has a subtropical maritime monsoon climate. Mean annual precipitation is 1768 mm. Mean annual temperature is 17.7°C, and monthly mean temperature ranges from 6.8°C in January to 28°C in July. The soil is red, with rich soil humus contents. The plantations were built and authorized by Research Institute of Forestry, Chinese Academy of Forestry. The field studies did not involve endangered or protected species.

### Biomass

Three stands of 7-, 16- and 28-year-old Chinese fir were selected for the investigation. Each plot comprised an area of 20 m×30 m and a buffer zone of similarly treated trees surrounded each plot. The tree diameter and height measurements in all of the plots were conducted after the tree height reached 1.3 m (Table 1). The trees were distributed in diameter classes of 6, 8, 10, …, 28. Diameter classes of 7-year-old stand range from 6 to 16, 6 to 22 of 16-year-old stand, and 8 to 28 of 28-year-old stand. One or two trees in each diameter class were destructively sampled. A total 39 trees were sampled. After the tree was felled, the fresh weights of stem wood, branch, foliage and root were measured, and the subsamples were selected and weighed on a portable digital balance in the field. After removal to the laboratory, each subsample was oven-dried to constant weight at 105°C to determine the proportion of dry biomass in each component. According to the ratio of dry weight to fresh weight, each compartment biomass was computed. The statistics of total biomass of the 39 trees was showed in Table1.

**Table 1:** Sample trees of Chinese fir plantation with different ages.

Attributes	7 year-old (n = 9 trees)			16-year-old(n = 14 trees)			28-year-old(n = 16 tress)		
	D(cm)	H(m)	Biomass(kg)	D(cm)	H(m)	Biomass(kg)	D(cm)	H(m)	Biomass(kg)
Min	5.7	4.9	5.76	5.6	5.9	6.82	8.7	10.3	13.61
Max	16.3	9.3	60.40	22.5	14.8	111.28	28	22.7	267.73
Mean	10.97	7.28	29.71	14.19	11.48	54.16	16.87	17.11	92.22
Std	3.84	1.61	20.16	5.28	2.70	34.88	5.85	3.34	18.24

Note: Std = standard deviation.

## Methods

### Biomass Model

In this study, we modeled tree biomass *W* (in kg) as a function of height *H* (measured in meters) and diameter *D* (in cm) with the allometric equation 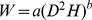. It is convenient to take logarithms for fitting model and dealing with heterocedasticity [28].
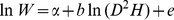
(1)where 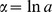 and *b* are the parameters of the model, and *e* is error term, which is normally distributed with mean zero and variance 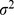.

### Bayes Rule

Let *y* = (*y*_1_, *y*_2_, *y*_3_, …) represent a vector of data and *θ* = (*θ*_1_, *θ*_2_, *θ*_3_, …) be a vector of parameters to be estimated. Bayes’ rule is then expressed as:
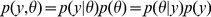
(2)where *p* represents the probability distribution or density function. Values for *θ* can be obtained by minimum least squares (MLS) or maximum likelihood estimation (MLE) in the classical approach. In the Bayesian framework, it uses probability distributions to describe uncertainty in the parameters being estimated. In light of the observed data, *θ* has a probability distribution given by:
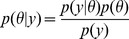
(3)We should note that the conditional distribution of θ given data y (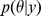) is what we are interested in estimating and is referred to the posterior probability distribution (simply called posterior) in the Bayesian framework. p(y | θ) tells us the distribution of y assuming θ is known, which is a likelihood function when regarded as a function of the parameters [14]. p(θ) is called the prior probability distribution for the parameters (simply called prior), and reflects information available about the hypothesis. The important characteristic of Bayesian method is that the parameters are treated as random variables [15], [25]. This is a very different assumption from that of classical method, which treats parameters as true, fixed (if unknown) quantities [14], [29].

In the study, the allometric relationship between tree biomass *W* and its diameter *D*, height *H* is given by a statistical model:
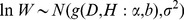
(4)where 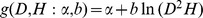 is the log of the allometric formula and gives the mean of the distribution of log-biomass. So Eqn (2) in the study can be said by
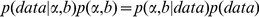
(5)where the data consist of triples (ln(W), D, H) measured from trees. In the current study, 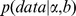 is the likelihood implied by Eqn (6):
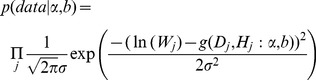
(6)


### Prior Distribution Specification

The choice of prior distribution is critical for Bayesian method [30]. In the above model specification, we need to choose appropriate prior distributions for all parameters, including 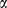, *b*. Many researchers choose to use non-informative normal (Gaussian) priors that reflect prior ‘ignorance’, which would not have a strong influence on the parameters. Such priors typically arise in the form of a parametric distribution with large or infinite variance. Alternatively, if prior information is available from external knowledge (reported parameters from the literature), the information can be used to construct a prior distribution. In this study, two prior distribution specifications were used in the Bayesian framework. One is non-informative prior, the other is informative prior. For non-informative prior, Gaussian priors on all parameters (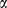, *b*) were *α*∼*N* (0, 1000), *b*∼*N* (0, 1000). For informative prior, we assumed the parameters of 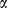, and *b* distributed as a bivariate normal distribution 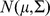, where 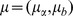 is a vector of means, and 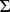 is the covariance matrix. The parameters of 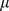 and 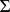 were specified from the reported literature. 32 biomass equations of Chinese fir (Table S1 in file S1) were collected from published references (Appendix S2 in file S1). The database has wide geographical distribution of southern China (Fig. 1). In addition, assuming that the errors are normally distributed 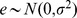, as recommended by Hadfield [31], the scalar parameters of the prior inverse Wishart of 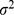 were set to *V* = 1 and *v* = 0.001.

The Bayesian method was implemented using the R package MCMCglmm [31] to fit the linear Gaussian model. MCMCglmm uses Gibbs sampling [32] to update the parameters. We set 25 000 iterations to run to ensure the obtainment of maximum convergence and satisfied posterior distributions of estimated parameters. To reduce the correlation between neighbouring iterations, the thinning parameters were all set to 3.

### Model Evaluation

Bayesian method was evaluated against the classical method (MLS), based on the following criteria. Smaller values of the criteria indicate that a model is better.
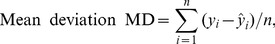
(7)

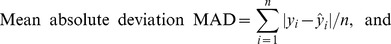
(8)

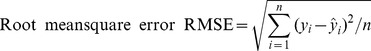
(9)where *y_i_* = observed values of tree biomass for the *i* ^th^ observation, 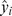 = predicted values of *y_i_*, 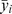 = mean values of *y_i_*, and *n* = number of observations.

## Results

**Figure 2:**
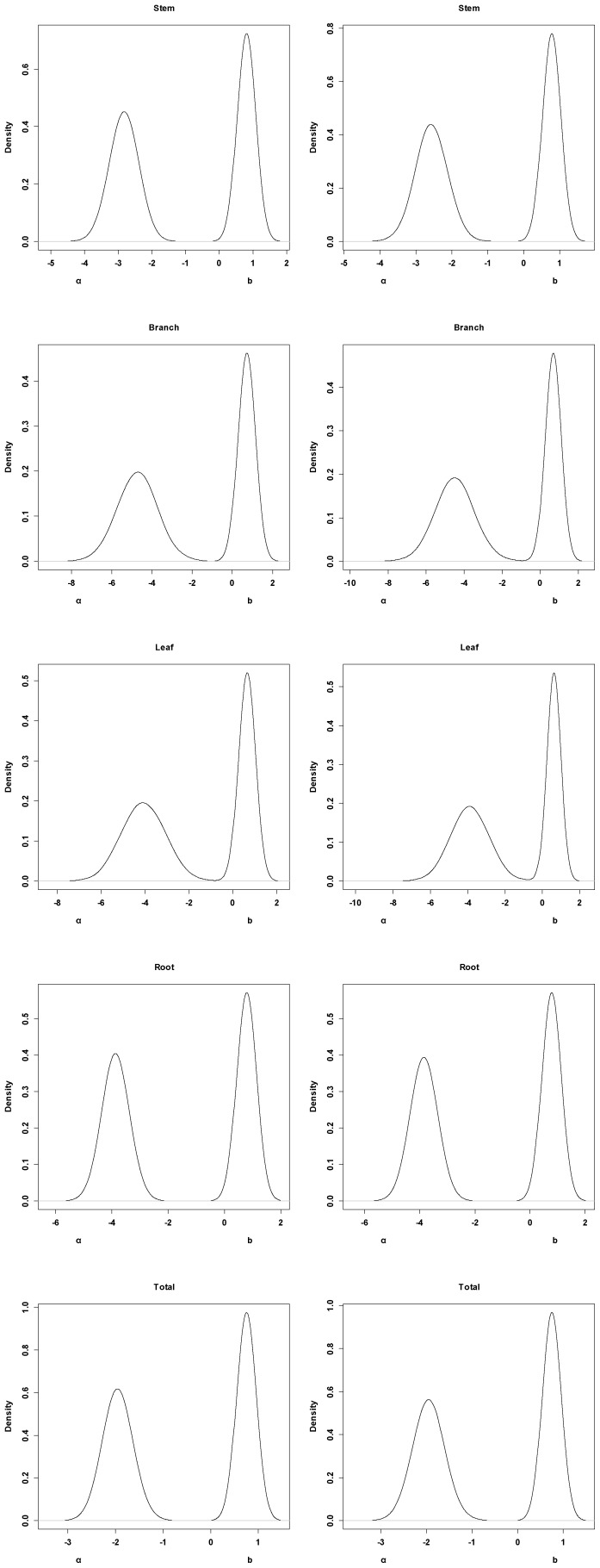
Posterior probability density of two parameters for each component biomass model. The left line is Bayesian method with informative prior, and the right line is Bayesian method with non-informative prior.

A total of 32 biomass equations in logarithmic form of Chinese fir were compiled from the reported literature for informative prior distributions. The parameters of 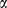, and *b* in each component biomass model distributed as a bivariate normal distribution (Table 2). In addition, we found that the two parameters are negatively correlated with each other. Based on the Bayesian method, the posterior probability distributions of the two parameters for each component biomass model were obtained. The posterior probability distributions were very similar based on Bayesian method with informative priors and non-informative priors (Fig. 2). Estimate values of 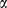 and *b* using the Bayesian with non-informative prior and MLS method were numerically identical in each component biomass model. The intervals of the two parameters estimates also had similar range, while they were wider than the interval from Bayesian method with informative prior (Table 3).

**Table 2:** Prior distribution of parameters in each component biomass equation of published literature.

Component	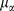	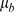	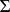
Stem	−3.8205	0.9270	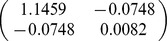
Branch	−5.8277	0.9136	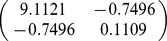
Foliage	−5.4356	0.8798	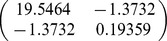
Root	−4.1500	0.8117	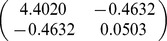
Total	−2.1133	0.8197	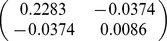

**Table 3:** Parameter estimates and 95% credible and confidence intervals of each component biomass model based on Bayesian method and MLS method.

Attributes	Stem	Branch	Foliage	Root	Total
Bayesian with informative prior					
*α*	−2.8305	−4.7061	−4.0269	−3.8680	−1.9488
	(−3.4976, −2.1275)	(−6.4565, −2.7886)	(−5.9187, −2.1755)	(−4.5988, −3.1633)	(−2.4496, −1.4794)
*b*	0.8067	0.7101	0.6441	0.7839	0.7493
	(0.7195, 0.8952)	(0.4562, 0.9291)	(0.4116, 0.8877)	(0.6944, 0.8782)	(0.6889, 0.8141)
Bayesian with non-informative prior					
*α*	−2.5721	−4.4901	−3.8780	−3.8516	−1.9500
	(−3.2987, −1.7868)	(−6.4075, −2.5373)	(−5.7382, −1.9090)	(−4.5875, −3.1354)	(−2.5230, −1.3568)
*b*	0.7734	0.6823	0.6242	0.7817	0.7489
	(0.6705, 0.8661)	(0.4339, 0.9363)	(0.3547, 0.8540)	(0.6864, 0.8736)	(0.6728, 0.8235)
MLS					
*α*	−2.5667	−4.4890	−3.8892	−3.8546	−1.9492
	(−3.3335, −1.7998)	(−6.4429, −2.5351)	(−5.8329, −1.9456)	(−4.5937, −3.1156)	(−2.5307, −1.3678)
*b*	0.7725	0.6819	0.6257	0.7823	0.7489
	(0.6737, 0.8713)	(0.4302, 0.9336)	(0.3753, 0.8761)	(0.6871, 0.8775)	(0.6740, 0.8238)

Evaluation statistics of Bayesian method and MLS method for biomass model were showed in Table 4. In stem biomass model, MD, MAD, and RMSE of Bayesian method with informative priors were the smallest among the three methods. The same results were found in branch biomass model, foliage biomass model, root biomass model, and total biomass model. RMSE is a widely accepted criterion for evaluating performance of a model. In the five models, compared with MLS method, Bayesian method with informative priors lowered RMSE ranging from 0.32% to 2.77%. Both each component biomass model and total biomass model, Bayesian method with informative priors was better than non-informative priors and MLS method. Bayesian method with non-informative priors was slightly better than MLS method in trunk biomass model, branch biomass model and leaf biomass model, while slightly worse in root biomass model and total biomass model.

**Table 4:** Evaluation statistics of Bayesian method, and MLS method for biomass model.

	Stem	Branch	Foliage	Root	Total
Bayesian with informative prior					
MD	0.7901	1.2294	1.0739	0.5986	1.7871
MAD	8.5600	2.1463	2.0769	2.6364	10.9544
RMSE	17.5511	3.8183	3.0433	3.4700	20.4366
Bayesian with non-informative prior					
MD	1.8751	1.2834	1.1316	0.6318	2.0543
MAD	8.6638	2.1905	2.1123	2.6423	10.9691
RMSE	17.8601	3.9227	3.0941	3.4853	20.5105
MLS					
MD	1.9693	1.2884	1.1273	0.6051	2.0193
MAD	8.6723	2.1923	2.1096	2.6404	10.9561
RMSE	17.8855	3.9272	3.0902	3.4757	20.5020

**Figure 3:**
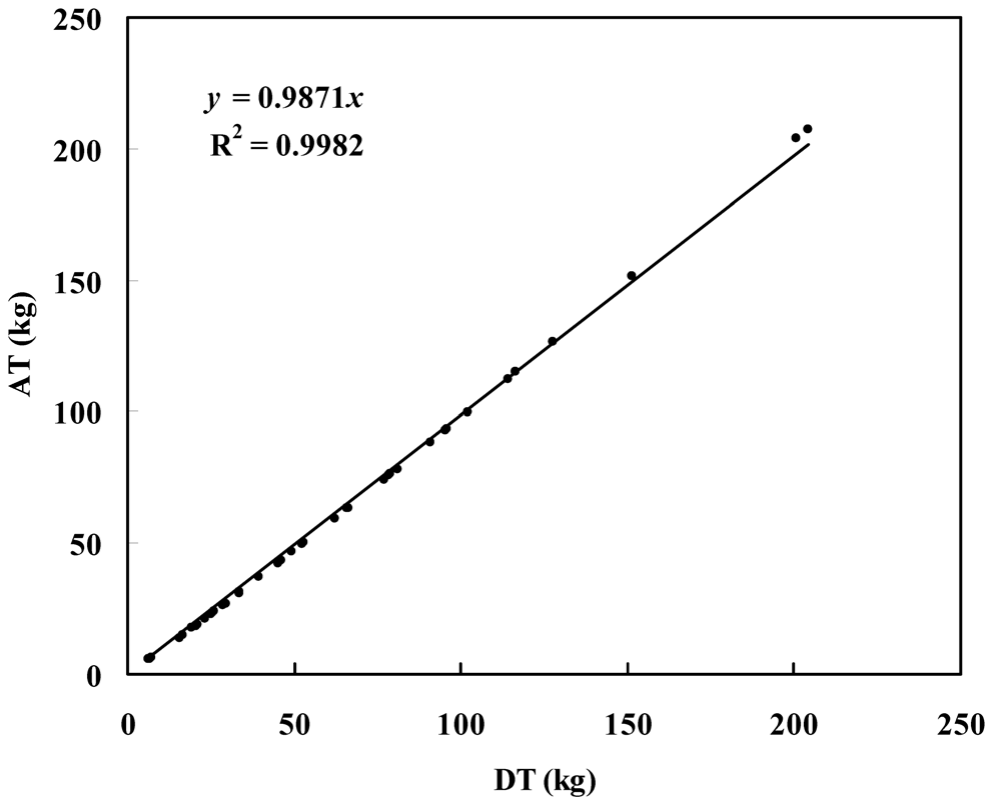
Correlation between total biomass estimates from summation of each component (AT) and direct regression of total biomass (DT).

In this study, each component biomass model and total biomass model were all developed. There were few biases between the total biomass estimates and summation of each component biomass estimates based on Bayesian method with informative priors (Fig. 3).

## Discussion

Chinese fir was one of the most important tree species for the biomass carbon pool in China from the 1980s to 2000s. The total biomass stock of Chinese fir increased continuously during the last three decades. The relative contribution of Chinese fir to the Chinese forest biomass stock increased from 2.48% in 1977–1981 to 4.32% in 1999–2003 [33]. The accurate estimation of biomass is critical for accounting and monitoring Chinese fir carbon stock. In this study, allometric model was used to model Chinese fir biomass. Over the past five decades, quite a few allometric equations have been used to estimate forest biomass [34], [35], [36], especially for two equations: 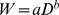, and 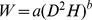. Previous studies have been reported for the estimation of Chinese fir biomass using the two equations with classical method [37], [38]. Although these two equations often give the impression of close relationships, good performance and high values of R^2^, they can still fail to get accurate estimates of stand biomass when they are applied to stands beyond the data range and site conditions [39]. Many different biotic and abiotic factors introduce variability in tree biomass model, suggesting that parameters of allometric equations are better represented by probability distributions (Fig. 2) rather than fixed values as classical method. Therefore, the widespread use of general Chinese fir biomass equations at the biome level obscures important differences in different stands.

In this study, we have presented a Bayesian solution to tree biomass models of Chinese fir. Bayesian method is an important statistical tool that is increasingly being used by ecologists. Bayes’ rule provides an alternative method for estimating parameters and expressing the degree of confidence or uncertainty in these estimates. Bayesian method allows for as much or as little data or prior knowledge and provides a direct measure of the probability of one or more hypotheses of interest [15]. Zapata-Cuartas et al. [27] found that model efficiency (RMSE) in the Bayesian method were almost identical to classical method when the sample size was larger than 60, and better when the sample size was smaller than 60. Here, the sample size was 39, and the model efficiency was better than classical method.

Bayesian credible interval and classical confidence are usually numerically identical if the Bayesian prior is non-informative [40]. This could be found in the study (Table 3). A non-informative prior is one in which the data (by the likelihood, which is *p*(*y* | *θ*) in Bayes’ rule) dominates the posterior, and the prior probabilities of all reasonable parameter values are approximately equal. Thus the posterior distribution has the same form as the likelihood. Since the posterior distribution with non-informative prior is less precise, the credible interval was wider and obtained worse prediction (Table 4).

Although Bayesian methods have been adopted in several applications in forestry, applications of Bayesian techniques in biomass are still relatively uncommon. In this study, a comprehensive sample of allometric equations of Chinese fir biomass gathered from published literature revealed that the parameters of allometric model can be well described by a bivariate normal distribution. The bivariate normal distribution (Table 2) was considered as prior distribution in Bayesian model for estimating tree biomass. It is the advantage of Bayesian method to update a model with priors. Therefore, not only are the data considered to be samples from a random variable, but the parameters to be estimated are regarded as random variables [15]. Thus, the performance of Bayesian method was better than classical method (Table 4).

It also notes that there are chances to improve the research. Additional variables can be included in the analysis and develop hierarchical Bayesian models that can yield more accurate priors for new data. For example, it would be possible to develop procedures in which the prior information adapts to specific site. For more comprehensive and accurate Chinese fir biomass model, additional variable describing site quality (e.g. site index) should be incorporated into the models [41]. We believe that the tree biomass modeling could be benefit from further explorations of the use of Bayesian method.

## Conclusions

Established over a broad geographical range, the national Chinese fir resource encompasses large variations in climatic, edaphic conditions, silviculture and genetic stock that affect biomass accumulation. These different biotic and abiotic factors introduce variability in Chinese fir biomass model, suggesting that parameters of allometric equations are better represented by probability distributions rather than fixed values. In Bayesian framework, appropriate prior distribution is very necessary. In this study, Bayesian methods with non-informative priors and informative priors were used to estimate Chinese fir biomass. For informative priors, 32 biomass equations of Chinese fir were collected from published references. The parameter distributions from published literature were considered as prior distribution in Bayesian model for estimating tree biomass. Bayesian method with non-informative prior and classical method got similar performance. The performance of Bayesian method with informative prior was better than non-informative prior and classical method, which provides a reasonable method for estimating Chinese fir biomass.

## Supporting Information

File S1
**Supporting Appendices.** Appendix S1. Parameter estimates of 32 biomass equations (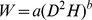) of Chinese fir collected from published literature. Appendix S1. Published literature estimating Chinese fir biomass with allometric equation.(DOC)

## References

[1] Wu Z (1984) Chinese Fir (in Chinese). Beijing: China Forestry Press.

[2] Lei J (2005) Chinese forest resources. China Forestry Publishing House, Beijing. (in Chinese).

[3] Hall DO (1997) Biomass energy in industrialised countries-a view of the future. For Ecol Manage 91: 17–45.

[4] Zianis D, Muukkonen P, Mäkipää R, Mencuccini M (2005) Biomass and stem volume equations for tree species in Europe. Silva Fenica Mongr 4: 1–63.

[5] Jenkins JC, Chojnacky DC, Heath LS, Birdsey RA (2003) National-scale biomass estimators for United States tree species. For Sci 49: 12–35.

[6] Lehtonen A, Mäkipää R, Heikkinen J, Sievänen R, Liski J (2004) Biomass expansion factors (BEFs) for Scots pine, Norway spruce and birch according to stand age for boreal forests. For Ecol Manage 188: 11–224.

[7] Lin S, Xu T, Zhou G (1991) Biomass of Chinese fir forest plantation (in Chinese). J Zhejiang Forestry College 8: 288–294.

[8] Tian D, Pan H, Kang W, Fang H (1998) A study of the biomass of a second generation Chinese fir plantation (in Chinese). J CSFU 18: 11–16.

[9] Holbrook NM, Putz FE (1989) Influence of neighbors on tree form: effects of lateral shade and prevention of sway on the allometry of Liquidambar styraciflua (sweet gum). Am J Bot 76: 1740–1749.

[10] King DA (1991) Tree allometry, leaf size and adult size in old growth forests of western Oregon. Tree Physiol 9: 369–381. 14972848 10.1093/treephys/9.3.369

[11] Chambers JQ, Santos JD, Ribeiro RJ, Higuchi N (2001) Tree damage, allometric relationships, and above-ground net primary production in central Amazon forest. For Ecol Manage 152: 73–84.

[12] Anholt BR, Werner E, Skelly DK (2000) Effect of food and predators on the activity of four larval ranid frogs. Ecology 81: 3509–3521.

[13] Shen TJ, Chao A, Liu CF (2003) Predicting the number of new species in further taxonomic sampling. Ecology 84: 798–804.

[14] Edwards D (1996) Comment: the first data analysis should be journalistic. Ecol Appl 6: 1090–1094.

[15] Ellison AM (2004) Bayesian inference in ecology. Ecol Lett 7: 509–520.

[16] Berger JO, Berry DA (1988) Statistical analysis and the illusion of objectivity. Am Sci 76: 159–65.

[17] Jaynes ET (2003) Probability Theory: The Logic of Science. Cambridge University Press, New York, USA.

[18] Green EJ, Jr. Roesch FAA, Smith FM, Strawderman WE (1994) Bayesian estimation for the three-parameter Weibull distribution with tree diameter data. Biometrics 50: 54–269.

[19] Bullock BP, Boone EL (2007) Deriving tree diameter distributions using Bayesian model averaging. For Ecol Manage 242: 127–132.

[20] Clark JS, Wolosin M, Dietze M, Ibáňez I, Ladeau S, et al (2007) Tree growth inference and prediction from diameter censuses and ring widths. Ecol Appl 17: 1942–1953. 17974333 10.1890/06-1039.1

[21] Wyckoff P, Clark JS (2000) Predicting tree mortality from diameter growth: a comparison of maximum likelihood and Bayesian approaches. Can J For Res 30: 156–167.

[22] Metcalf CJE, McMahon SM, Clark JS (2009) Overcoming data sparseness and parametric constraints in modeling of tree mortality: a new nonparametric Bayesian model. Can J For Res 39: 1677–1687.

[23] Green EJ, Valentine HT (1998) Bayesian analysis of the linear model with heterogeneous variance. For Sci 44: 134–138.

[24] Green EJ, Strawderman WE (1996) Predictive posterior distributions from a Bayesian version of a slash pine yield model. For Sci 42: 456–464.

[25] Li R, Stewart B, Weiskittel A (2012) A Bayesian approach for modelling non-linear longitudinal/hierarchical data with random effects in forestry. Forestry 85: 17–25.

[26] Nyström K, Ståhl G (2001) Forecasting probability distributions of forest yield allowing for a Bayesian approach to management planning. Silva Fennica 35: 185–201.

[27] Zapata-Cuartas M, Sierra CA, Alleman L (2012) Probability distribution of allometric coefficients and Bayesian estimation of aboveground tree biomass. For Ecol Manage 277: 173–179.

[28] Overman JPM, Witte HJL, Saldarriaga JG (1994) Evaluation of regression models for above-ground biomass determination in Amazon rainforest. J Trop Ecol 10: 207–218.

[29] de Valpine P, Hastings A (2002) Fitting population models incorporating process noise and observation error. Ecol Monogr 72: 57–76.

[30] Gelman A, Carlin JB, Stern HS, Rubin DB (2004) Bayesian Data Analysis, 2nd edn. Boca Raton, FL, USA: Chapman and Hall/CRC.

[31] Hadfield JD (2010) MCMC methods for multi-response generalized linear mixed models: the MCMCglmm R package. J Stat softw 33: 1–22.20808728

[32] Chib S, Greenberg E (1995) Understanding the Metropolis-Hastings algorithm. Am Stat 49: 327–335.

[33] Wang B, Wei W, Xing Z, You W, Niu X, et al (2012) Biomass carbon pools of Cunninghamia lanceolata (Lamb.) Hook. forests in subtropical China: Characteristics and potential. Scan J Forest Res 27: 545–560.

[34] Hui G (1989) A study on the productivity of common, China fir (Cunninghamia lanceolata) plantation at hilly area in Dagang Mountain, Jiangxi province. Scientia Silvae Sinicae25: 564–569 (in Chinese)..

[35] Ter-Mikaelian MT, Korzukhin MD (1997) Biomass equations for sixty-five North American tree species. For Ecol Manage 97: 1–24.

[36] Basuki TM, van Laake PE, Skidmore AK, Hussin TA (2009) Allometric equations for estimating the above-ground biomass in tropical lowland Dipterocarp forests. For Ecol Manage 257: 1684–1694.

[37] Li S, Shi J, Wu H (2007) Biomass and vertical distribution of the second-growth Chinese fir plantation. For Eng 23: 1–4 (in Chinese)..

[38] Liu W, Xiang W, Tian D, Yan W (2010) General allometric equations for estimating Cunninghamia lanceolata tree biomass on large scale in southern China. J CSUFT 30: 7–14 (in Chinese)..

[39] Case BS, Hall RJ (2008) Assessing prediction errors of generalized tree biomass and volume equations for the boreal forest region of west-central Canada. Can J For Res 38: 878–889.

[40] McCarthy MA (2007) Bayesian methods for ecology. Cambridge University Press, Cambridge, UK.

[41] Bi H, Long Y, Turner J, Lei Y, Snowdon P, et al (2010) Additive prediction of aboveground biomass for *Pinus radiate* (D. Don) plantations. For Ecol Manage 259: 2301–2314.

