# Supplementary material for: Tree Biomass Estimation of Chinese fir (Cunninghamia lanceolata) Based on Bayesian Method
Source: PLoS One. 2013 Nov 20;8(11):e79868. doi: 10.1371/journal.pone.0079868 (PMC3835933; doi:10.1371/journal.pone.0079868)
Supplement: File S1 — Supporting Appendices. Appendix S1. Parameter estimates of 32 biomass equations () of Chinese fir collected from published literature. Appendix S1. Published literature estimating Chinese fir biomass with allometric equation. (DOC) [file pone.0079868.s001.doc]

**Appendix S1**

Parameter estimates of 32 biomass equations ($W=a(D^{2}H)^{b}$) of Chinese fir collected

from published literature.

| No. | *a* | | | | | *b* | | | | | Reference |
| --- | --- | --- | --- | --- | --- | --- | --- | --- | --- | --- | --- |
|  | Stem | Branch | Foliage | Root | Total | Stem | Branch | Foliage | Root | Total |  |
| 1 | 0.0388 | 0.0090 | - | 0.0597 | - | 0.9161 | 1.1812 | - | 0.6838 | - | [1] |
| 2 | 0.0085 | 0.0017 | - | 0.0804 | - | 1.1072 | 1.0919 | - | 0.6462 | - | [1] |
| 3 | 0.02 | 0.001 | 0.0347 | 0.002 | - | 0.9134 | 1.0445 | 0.6297 | 1.0561 | - | [2] |
| 4 | 0.0163 | 0.3813 | 0.0058 | 0.0436 | - | 0.9582 | 0.3344 | 0.889 | 0.6464 | - | [3] |
| 5 | 0.2278 | 0.0008 | 0.0122 | 0.003 | - | 0.7057 | 1.1087 | 0.6916 | 0.9834 | - | [4] |
| 6 | 0.2645 | 0.0120 | 0.0386 | 0.1359 | - | 0.7034 | 0.8550 | 0.6412 | 0.5896 | - | [4] |
| 7 | 0.0217 | - | - | - | - | 0.9417 | - | - | - | - | [5] |
| 8 | 0.01 | 1.223 | 0.8511 | - | 0.0707 | 1.0366 | 0.2573 | 0.2587 | - | 0.8543 | [6] |
| 9 | 0.0205 | 0.0257 | 0.0226 | 0.0354 | 0.0603 | 1.0105 | 0.7265 | 0.7817 | 0.788 | 0.9912 | [7] |
| 10 | 0.084 | 0.00005 | 0.1152 | 0.0097 | - | 0.7902 | 0.5843 | 0.5308 | 0.8593 | - | [8] |
| 11 | 0.0341 | 0.00001 | 0.0205 | 0.0326 | - | 0.8619 | 0.733 | 2.1448 | 0.7271 | - | [8] |
| 12 | 0.0008 | 0.0005 | 1.4E-7 | 0.0069 | - | 1.0271 | 1.1755 | 1.2558 | 1.0166 | - | [8] |
| 13 | 0.0321 | 0.0032 | 2.7E-9 | 3.7E-6 | - | 0.8919 | 0.9631 | 1.7464 | 1.6733 | - | [8] |
| 14 | 0.0032 | 0.006 | 0.0043 | 0.0091 | - | 0.8631 | 1.0781 | 0.9075 | 0.8045 | - | [8] |
| 15 | 0.0183 | 0.0024 | 0.0028 | 0.0121 | - | 0.9663 | 0.9591 | 0.9815 | 0.8371 | - | [9] |
| 16 | 0.0734 | 0.0005 | 0.0196 | 0.0431 | - | 0.8626 | 1.2331 | 0.7897 | 0.7358 | - | [10] |
| 17 | 0.0163 | 0.0028 | 0.0058 | 0.0436 | 0.119 | 0.9958 | 1.0036 | 0.889 | 0.931 | 0.791 | [11] |
| 18 | 0.0402 | 0.1802 | 0.4082 | 0.0538 | 0.2236 | 0.83 | 0.4307 | 0.2944 | 0.7004 | 0.6912 | [12] |
| 19 | 0.0323 | 0.0256 | 0.0865 | 0.0429 | 0.1495 | 0.8924 | 0.6774 | 0.5067 | 0.7001 | 0.7592 | [13] |
| 20 | 0.025 | 0.012 | 0.009 | 0.014 | - | 0.935 | 0.821 | 0.77 | 0.844 | - | [14] |
| 21 | 0.0151 | 0.0127 | 0.0907 | 0.0106 | - | 0.9564 | 0.8026 | 0.5561 | 0.8344 | - | [15] |
| 22 | 0.0287 | 0.0023 | 0.0119 | 0.0444 | - | 0.8674 | 1.0152 | 0.8256 | 0.6596 | - | [16] |
| 23 | 0.0097 | 0.0018 | 0.0028 | - | - | 0.9991 | 0.99994 | 0.9995 | - | - | [17] |
| 24 | 0.0099 | 0.0014 | 0.0016 | - | - | 0.9994 | 0.9999 | 0.99997 | - | - | [17] |
| 25 | 0.0103 | 0.0009 | 0.0007 | - | - | 0.9999 | 0.99996 | 0.9999 | - | - | [17] |
| 26 | 0.0298 | - | - | - | - | 0.8897 | - | - | - | - | [18] |
| 27 | 0.0285 | - | - | - | - | 0.8937 | - | - | - | - | [18] |
| 28 | 0.0230 | - | - | - | - | 0.9119 | - | - | - | - | [18] |
| 29 | 0.0295 | - | - | - | - | 0.8805 | - | - | - | - | [18] |
| 30 | 0.037 | 0.0038 | 0.0186 | - | - | 0.8808 | 0.9434 | 0.7089 | - | - | [19] |
| 31 | 0.0069 | 2.67E-7 | 1.57E-6 | 0.0336 | - | 1.0802 | 2.0305 | 1.7632 | 0.7073 | - | [20] |
| 32 | 0.0285 | 0.0367 | 0.127 | 0.0436 | 0.1836 | 0.9576 | 0.6167 | 0.4343 | 0.7172 | 0.8311 | [21] |

Note: References were showed in Appendix B.

**Appendix S2**

Published literature estimating Chinese fir biomass with allometric equation.

1. Ai X, Zhou G (1996) Study on the biomass of Chinese fir plantation’s ecosystem in the north edge of median sub-tropic. Hubei For Sci Technol 2: 17-20 (in Chinese).
2. Cai X, Zhang Z, Ouyang X, Zhang Q (1997) A study on China fir plantation biomass. Acta Agriculturae Universities Jiangxiensis 19: 138-145 (in Chinese)
3. Fang X, Tian D (2006) Dynamic of carbon stock and carbon sequestration in Chinese fir plantation. Guihaia 26: 516-522 (in Chinese).
4. Fang X, Tian D, Xiang W (2010) Effects of thinning on carbon storage and its spatial distributions in Chinese fir plantation ecosystem. J CSUFT 30: 47-53 (in Chinese).
5. Fu X (2000) Effects of the different thinning intensity of *cunninghamia lanceolata* forests on the stand biomass. J Fujian For Sci and Tech 27: 41-43 (in Chinese).
6. Hu Y, Pang Q (2012) Study on the Biomass and Distribution Pattern of *Cunninghamia lanceolata*(Lamb.)Hook. in Western of Hubei. Hubei For Sci Technol 3: 6-9 (in Chinese).
7. Huang X, Wu C, Hong W, Li Z, Chen Z (2011) The relationship between stand density and biomass of two rotation Chinese fir plantations. J Fujian College of Forestry 31: 102-105 (in Chinese).
8. Huang Z, Tian D, Kang W, Xiang W, Yan W (2011) Dynamics of biomass distribution in first rotation of Chinese fir plantations in Huitong County, Hunan Province. J CSUFT 31: 37-43 (in Chinese).
9. Hui G (1989) A study on the productivity of common, China fir (Cunninghamia lanceolata) plantation at hilly area in Dagang mountain, Jiangxi province. Scientia Silvae Sinicae, 1989, 25: 564-569 (in Chinese).
10. Li H, Lei Y (2010) Estimation and evaluation of forest biomass carbon storage in China. Chins Forestry Publishing House, Beijing (in Chinese).
11. Li S, Shen C (2006) The spatial-temporal characters of biomass in the secondary generation of Chinese fir plantation. Forest Engineering, 22 (1): 5-7 (in Chinese)
12. Lin S, Xu T, Zhou G (1991) Biomass of Chinese fir forest plantation. J Zhejiang Forestry College 8: 288-294 (in Chinese).
13. Liu W, Xiang W, Tian D, Yan W (2010) General allometric equations for estimating *Cunninghamia lanceolata* tree biomass on large scale in southern China. J CSUFT 30: 7-14 (in Chinese).
14. Shen Y, Tian D, Yan W, Xiao Y (2011) Biomass and its distribution of natural secondary *Quercus fabric* + *Sassafras tsumu* + *Cunninghamia lanceolata* community in Yuanling County, Hunan Province. J CSUFT 31: 45-51 (in Chinese).
15. Tian D, Pan H, Kang W, Fan H (1998) A study of the biomass of a second generation Chinese fir plantation. J CSUFT 18: 11-16 (in Chinese).
16. Tian D, Xiang W, Yan W, Kang W (2002) Effect of successive-rotation on productivity and biomass of Chinese fir plantation at fast growing stage. Scientia Silvae Sinicae 38: 14-18 (in Chinese).
17. Tong J (2008) Study on the biomass productivity and growth of Chinese fir plantations of different site and density. J FAFU(Natural Science Edition) 37: 369-373 (in Chinese).
18. Wen Y, Liang Q, Jiang H (1995) Study on the biomass and distribution law of Chinese fir plantation in Guangxi. J Guangxi Agricultural University 14: 55-64 (in Chinese).
19. Zhang L, Huang Y, Luo T, Dai Q, Deng K (2005) Age effects on stand biomass allocations to different components: a case study in forests of *Cunninghamia lanceolata* and *Pinus Massoniana*. J Graduate School of the Chinese Academy of Sciences 22: 70-178 (in Chinese).
20. Zhao K, Tian D (2000) Study of the Biomass and Productivity of Mature Chinese Fir Stand in Huitong County. J CSUFT 20: 8-13 (in Chinese).
21. Zhou G, Yao J, Qiao W, Yang Q, Zhu G, Xu W (1996) Biomass of Chinese fir planted forest in Qinyuan of Zhejiang. J Zhejiang Forestry College 13: 235-242 (in Chinese).
